# Long-Term Results of Reconstruction with Pelvic Allografts after Wide Resection of Pelvic Sarcomas

**DOI:** 10.1155/2014/605019

**Published:** 2014-01-27

**Authors:** Mehmet Ayvaz, Senol Bekmez, M. Ugur Mermerkaya, Omur Caglar, Emre Acaroglu, A. Mazhar Tokgozoglu

**Affiliations:** ^1^Department of Orthopaedics and Traumatology, Faculty of Medicine, Hacettepe University, 06100 Ankara, Turkey; ^2^Department of Orthopaedics and Traumatology, Dr. Sami Ulus Training and Research Hospital, 06100 Ankara, Turkey; ^3^Department of Orthopaedics and Traumatology, Faculty of Medicine, Bozok University, 66000 Yozgat, Turkey; ^4^Ankara Spine Center, Iran Street 45/2 Kavaklidere, 06100 Ankara, Turkey

## Abstract

Reconstruction after the resection of a pelvic tumor is a challenging procedure in orthopedic oncology. The main advantage of allograft reconstruction is restoration of the bony architecture of the complex pelvic region. However, high complication rates such as infection and allograft resorption had been reported in the literature. In this study, we aimed to retrospectively review nine patients treated with pelvic resection and structural pelvic allograft reconstruction. Functional results, complications, and survival of the patients and the allografts were evaluated. At a mean follow-up of 79 months, three patients were dead. Major complications were detected in eight of the nine patients. Infection (four of the nine patients) and allograft resorption (three of the nine patients) were the most common causes of failure. The cumulative survival of the patients was 66.7 percent at 70 months. However, allograft survival was only 26.7 percent at 60 months. Mean MSTS score was 69. In conclusion, we suggest that other reconstruction options should be preferred after pelvic resections because of the high complication rates associated with massive allograft reconstruction.

## 1. Introduction

Treatment of sarcomas of the pelvis remains to be a challenge due to high rates of complications such as local recurrence, infection, and mortality. Since there are no true compartments unlike extremities, a delay in diagnosis, and close proximity to vital intrapelvic structures, it is not always possible to reach wide surgical margins. Traditionally, radical amputations had been the only surgical modality in the treatment of pelvic malignant tumors [1]. Improvements in neoadjuvant chemotherapy and imaging modalities made limb-sparing surgery feasible in orthopedic oncology [2, 3]. Later this experience was transferred to treatment of sarcomas of the pelvis. Reconstruction options after resection of a pelvic sarcoma are prosthesis and cement [4], “Saddle" prosthesis [5], reimplantation of the resected bone after autoclaving [6, 7] or irradiating [8], proximal femoral autograft [9] and pelvic allografts [10–13]. Although pelvic allograft reconstruction restores the complex bony architecture of the pelvic region, high rates of infection and mechanic failure had been reported [11, 14]. In this study, we retrospectively evaluated the functional results, complications, and survival analysis of nine patients with wide resection of a sarcoma in the pelvic region and allograft reconstruction with or without a total hip replacement.

## 2. Patients and Methods

### 2.1. Study Population

Nine consecutive patients (7 males, 2 females) with a mean age of 22 (range from 12 to 52) from 2000 to 2007, who underwent a wide pelvic resection and were reconstructed with a fresh-frozen hemipelvic allograft with or without cemented total hip prosthesis, were retrospectively evaluated for this study. The average follow-up was 79 (range 13 to 118) months. All had a diagnosis of malignant sarcoma of the pelvis (Table 1). After appropriate diagnostic evaluation, the patients were graded with the Enneking Musculoskeletal Tumor Grading System. Eight patients were Enneking grade IIB and one patient was Enneking grade 3. The surgical resections were classified by using the Enneking et al. classification [15]. For postoperative functional evaluation, the revised Musculoskeletal Tumor Society (MSTS) Rating Scale was used [16]. Allograft resorption was classified according to the classification system proposed by the Mount Sinai Hospital in Toronto; mild resorption is defined as partial-thickness resorption of less than one centimetre in width and length, moderate resorption is defined as partial-thickness resorption of more than one centimetre in width and length, and severe resorption is defined as full-thickness resorption of any length [17].

Survival analysis of the patients, allografts, and reconstructions were evaluated with the Kaplan Meier survival analysis using the SPSS statistical package (version 20.0; SPSS, Chicago, IL).

### 2.2. Surgical Technique

The patients were operated in a supine position utilizing an extended iliofemoral approach. After securing the neurovascular bundle, the sarcomas were excised with a wide surgical margin. After resection, the defects were reconstructed with a fresh-frozen hemipelvis allograft (Table 2). The allografts were initially thawed in a saline solution and then shaped to the defect. Following this, the graft was rigidly fixed using pelvic reconstruction plates and cancellous screws. After rigid fixation of the allograft, the acetabulum of the allograft was reamed and a cemented polyethylene acetabular component was inserted and followed by insertion of a femoral component in six patients for whom an extra-articular resection was done. The proximal femur was preserved in the remaining three patients for whom an intra-articular resection was performed.

## 3. Results 

Average follow-up of the patients was 79 (range from 13 to 118) months. At the latest follow-up, three patients were expired. They expired at the 13th, 36th, and 71st months, consequently. Two early deaths were related to local recurrence or dissemination of the sarcoma. One death was related to sepsis caused by failure to suppress the deep infection. Six patients were alive at the latest follow-up and five of them were disease free. Patient data is summarized in Table 3.

### 3.1. Complications

**Figure 1:**
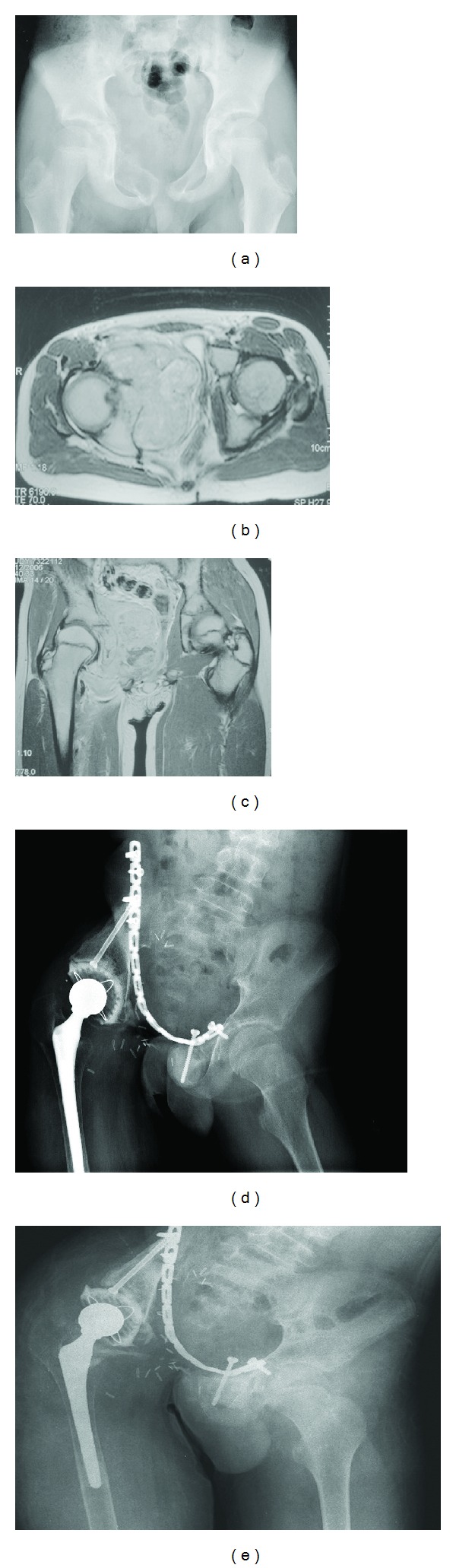
A twelve-year-old male with a diagnosis of Ewing sarcoma in the pelvic region (a). Axial (b) and coronal (c) magnetic resonance images at diagnosis. Type II-III internal hemipelvectomy and reconstruction with alloprosthetic composite were performed (d). After 62 months, severe allograft resorption and purulent drainage were detected in the surgical site (e). External hemipelvectomy was performed for the recalcitrant infection. The patient was dead because of sepsis in the 71st month.

Complication rates in our series were high (in eight of the nine patients). Only one patient was complication-free. Infection was the most important cause of complication and failure. Four of the nine patients had an infection at the surgical site. Multiple debridements were done to control the infection. Infection led to allograft and implant removal with local antibiotic impregnated bead insertion in two cases. One patient underwent a two-staged revision and reconstruction with custom-made pelvis prosthesis. An antibiotic impregnated cement spacer was implanted during the first stage. In one patient, the persistent infection led to a hemipelvectomy after multiple attempts of debridement. The infection and related complications eventually caused the death of this patient (Figure 1). Overall, two of four patients with infection expired.

Allograft resorption was observed in three of the nine patients. The grade of resorption in entire patients was severe according to the classification system determined by Haddad et al. [17]. In one of these patients, debridement and implant removal were performed due to pain. One patient with allograft resorption was revised with a pelvic allograft. One patient with allograft resorption was symptom free at the latest follow-up. Overall, seven of nine patients required reoperation.

### 3.2. Oncological Aspects

One patient with a marginal tumor resection zone had local recurrence and two other patients had distant metastases. The patient with local recurrence eventually underwent hemipelvectomy. The patient with local recurrence and another patient with distant metastases expired. One patient with Ewing sarcoma had a solitary distant metastasis on distal femur after eight years from the initial diagnosis. Wide resection and reconstruction with modular tumor resection prosthesis were performed. The remaining six patients were tumor-free at the latest follow-up.

### 3.3. Survival Analysis

**Figure 2:**
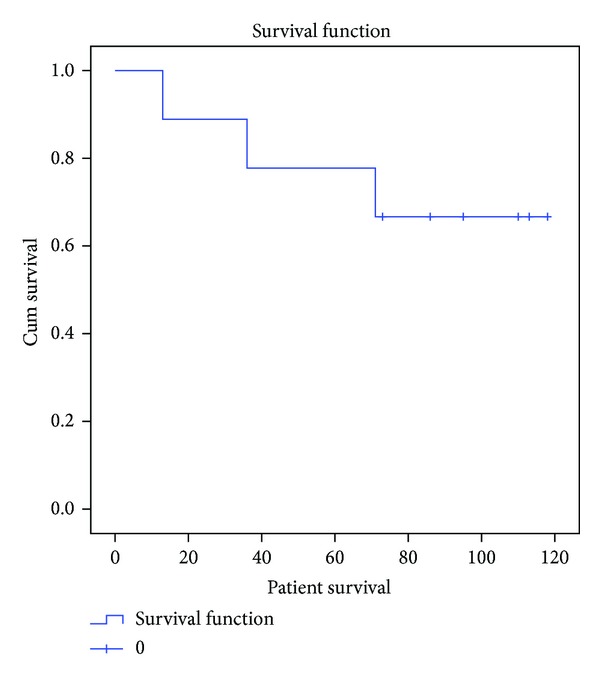
Kaplan-Meier analysis diagram demonstrating the overall cumulative survival of the patients.

**Figure 3:**
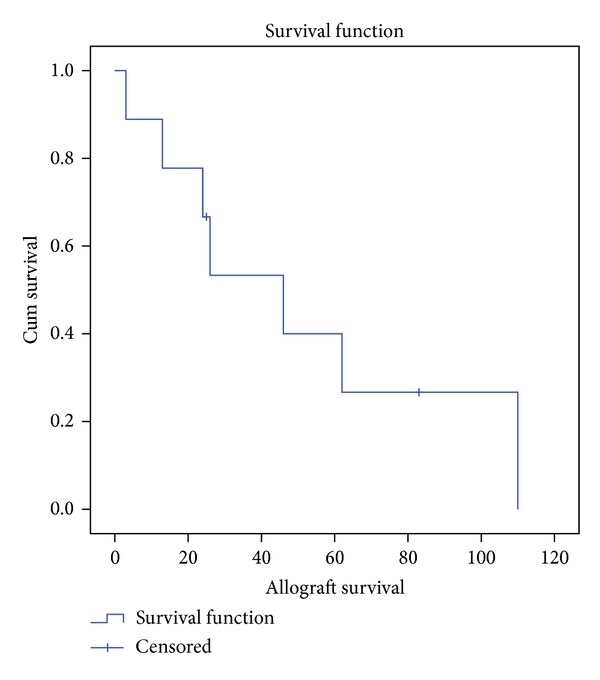
Kaplan-Meier analysis diagram demonstrating the allograft survival with the allograft removal as an endpoint.

Kaplan-Meier analysis demonstrated that overall cumulative patient survival was 66.7 percent at 70 months (Figure 2). With using allograft removal as an endpoint allograft survival was determined to be only 26.7 percent at 60 months (Figure 3).

### 3.4. Functional Analysis

Four of the nine patients were able to walk without external support. The remaining five patients could walk with one crutch on the opposite site. Mean MSTS score was 69 (54–87) after allograft reconstruction. Early functional results were better especially in the cases in which local and systemic disease control could have been achieved. However, multiple surgical interventions because of infection and allograft resorption caused gradual deterioration on functional outcome.

## 4. Discussion

Resection with wide margins and reconstruction of a pelvic sarcoma are a problematic procedure. Commonly, delay in diagnosis leading to a large tumor that invades major neurovascular structures makes such a procedure impossible in the pelvic region. Therefore, the prognosis of a pelvic sarcoma in a central localization is accepted as worse than a peripheral localization. Local recurrence is the major determinant of the prognosis in the aspect of long-term survival of the patients with sarcoma. In our series, we could not provide wide resection in one patient with a diagnosis of osteosarcoma in the index operation. Afterwards, the patient expired at an early period because of tumor dissemination despite an external hemipelvectomy being performed later. Conversely, six of the remaining eight patients for whom wide resection margins could have been achieved were disease-free at the latest follow-up. The early functional results were also better in the cases in which local and systemic disease control could have been achieved.

Resection of an isolated iliac/pubic lesion does not lead to mechanical instability, therefore not requiring a reconstruction. However in terms without reconstruction, the patients treated with periacetabular resections would need supportive devices for ambulation because of limb-length inequality, structurally and mechanically insufficient pelvic architecture. So, complex reconstructive procedures are necessary after periacetabular pelvic resections for function [6, 18]. Filling the dead space after pelvic tumor resection is a major problem [19]. Several surgical techniques have been proposed to salvage the limb after periacetabular resection. Surgical pseudoarthrosis, iliofemoral/ischiofemoral arthrodesis, filling the defect with bone cement and Steinmann pins, autoclaved autografts, vascularized fibular reconstruction, saddle-type prosthesis, and alloprosthetic combinations are included among these techniques. Because of reported high local complication rates and unsatisfactory functional results, there is no consensus on the best way for reconstruction after such a resection.

Reconstruction with massive allografts following wide resection of the pelvis has been advocated as an option. In earlier studies, good functional results were reported despite high mechanical failure and infection rates [20]. Advantages of the procedure are the possibility of early weight bearing, early mobilization, and satisfactory function when coupled with a hip prosthesis, restoration of the normal anatomy, and preservation of the extremity length [13, 21]. Cosmetically near-normal appearance is very important for the patient satisfaction. Another advantage of the procedure is the easy accessibility of the pelvic allografts in comparison with the custom-made metal pelvic prostheses. The main disadvantage of the allograft reconstruction is the high rates of complications (30 to 90 percent) such as local recurrence (9.6 to 33 percent) in the pelvic region despite wide resections, sciatic nerve palsy (up to 24 percent of cases), deep infections (up to 15 percent of cases), and instability (up to 20 percent) [11, 13, 19, 22]. More recent studies represent more satisfactory results in terms of functional outcomes and complication rates in selected patient populations reconstructed with fresh-frozen allografts [14, 21]. Nevertheless, in our series of nine patients, even though reconstructions were performed with fresh-frozen allografts, we encountered a high rate of complications despite functional scores similar to previous studies. The major causes of failure in our series were infection and allograft resorption that were so severe that they required multiple operations and allograft removal. Unfortunately, only one-quarter of the allografts could survive in long-term follow-up. Functional outcomes were also gradually deteriorated subsequent to the allograft failure. In other words, the complication rates and allograft survival in our series were not as satisfactory as previously reported in similar studies in the literature.

Despite high complication rates and surgical difficulties, we agree with the fact that reconstruction is imperative to improve the quality of life of the patients after wide resections of the pelvic tumors. We experienced higher rates of complications in our series in comparison to the literature.

## 5. Conclusion

Despite restoration of the normal anatomy of the pelvic region and good functional outcomes achieved, we do not recommend the use of allografts due to high complication rates. Common problems are infection, allograft resorption, local recurrence, and implant failure. So, we suggest that other reconstruction options such as custom made pelvic prosthesis should be considered.

## Figures and Tables

**Table 1:** Diagnosis and tumor grades.

Case	Diagnosis	Tumor grade
1	Osteosarcoma	IIB
2	Malignant fibrous histiocytoma	III
3	Postradiation sarcoma	IIB
4	Ewing sarcoma	IIB
5	Ewing sarcoma	IIB
6	Ewing sarcoma	IIB
7	Giant cell tumor	3
8	Chondrosarcoma	IIB
9	Osteosarcoma	IIB

**Table 2:** Type of resection and fixation.

Case	Resection type	Side	Resection zone	Allograft	THA
1	I-II	Right	Extra-articular	Fresh-frozen	Cemented
2	II-III	Left	Extra-articular	Fresh-frozen	Cemented
3	I-II	Left	Intra-articular	Fresh-frozen	—
4	I-II	Right	Extra-articular	Fresh-frozen	Cemented
5	I-II-III	Right	Intra-articular	Fresh-frozen	—
6	II-III	Right	Extra-articular	Fresh-frozen	Cemented
7	II-III	Right	Extra-articular	Fresh-frozen	Cemented
8	II-III	Right	Intra-articular	Fresh-frozen	—
9	I-II	Left	Extra-articular	Fresh-frozen	Cemented

**Table 3:** An overview of the patient data (Resec.: resection, Che: chemoteraphy, f/u: follow-up).

Case	Gender/ age at diagnosis	Tumor type	Grade	Resec. zone	THA	Operation time (hours)	Transfusion (mL)	Margins	Che	Complication	Tumor condition	Walking	MSTS score (%)	Latest f/u	Allograft survival (months)	Patient survival (months)	Reoperation
1	F/15	Osteosarcoma	IIB	I-II	+	5	7	Wide	+	Resorption Dislocation	No evidence of disease	Without support	74	No evidence of disease	46	113	Revision with allogreft
2	M/52	Malignant fibrous histiocytoma	III	II-III	+	5	6	Wide	−	Infection	Metastasis	1 crutch	54	Died at 13 months	3	13	Debridement Implant removal
3	F/13	Postradiation osteosarcoma	IIB	I-II	+	4	6	Marginal	+	—	Local recurrence	1 crutch	57	Died at 36 months	25	36	External hemipelvectomy
4	M/16	Ewing	IIB	I-II	+	5	7	Wide	+	Resorption	Metastasis	1 crutch	67	Distant metastasis	24	95	Debridement
5	M/26	Ewing	IIB	I-II-III	+	6	8	Wide	+	—	No evidence of disease	Without support	87	No evidence of disease	83	118	—
6	M/12	Ewing	IIB	II-III	+	6	7	Wide	+	Infection	No evidence of disease	1 crutch	72	Died at 71 months	62	71	External hemipelvectomy
7	M/25	Giant cell tumor	3	II-III	+	4	6	Wide	−	Infection	No evidence of disease	1 crutch	68	No evidence of disease	26	73	Debridement Implant removal
8	M/24	Chondrosarcoma	IIB	II-III	+	5	8	Wide	−	Infection	No evidence of disease	Without support	62	No evidence of disease	13	86	2-stage revision with custom made pelvis prosthesis
9	M/15	Osteosarcoma	IIB	I-II	+	5	8	Wide	+	Resorption	No evidence of disease	Without support	80	No evidence of disease	110	110	—
